# Long-Distance and Frequent Movements of the Flying-Fox *Pteropus poliocephalus*: Implications for Management

**DOI:** 10.1371/journal.pone.0042532

**Published:** 2012-08-03

**Authors:** Billie J. Roberts, Carla P. Catterall, Peggy Eby, John Kanowski

**Affiliations:** 1 School of Environment and Environmental Futures Centre, Griffith University, Nathan, Queensland, Australia; 2 School of Biological, Earth and Environmental Sciences, University of New South Wales, Sydney, New South Wales, Australia; 3 Australian Wildlife Conservancy, Malanda, Queensland, Australia; University of California, Berkeley, United States of America

## Abstract

Flying-foxes (Pteropodidae) are large bats capable of long-distance flight. Many species are threatened; some are considered pests. Effective conservation and management of flying-foxes are constrained by lack of knowledge of their ecology, especially of movement patterns over large spatial scales. Using satellite telemetry, we quantified long-distance movements of the grey-headed flying-fox *Pteropus poliocephalus* among roost sites in eastern Australia. Fourteen adult males were tracked for 2–40 weeks (mean 25 weeks). Collectively, these individuals utilised 77 roost sites in an area spanning 1,075 km by 128 km. Movement patterns varied greatly between individuals, with some travelling long distances. Five individuals travelled cumulative distances >1,000 km over the study period. Five individuals showed net displacements >300 km during one month, including one movement of 500 km within 48 hours. Seasonal movements were consistent with facultative latitudinal migration in part of the population. Flying-foxes shifted roost sites frequently: 64% of roost visits lasted <5 consecutive days, although some individuals remained at one roost for several months. Modal 2-day distances between consecutive roosts were 21–50 km (mean 45 km, range 3–166 km). Of 13 individuals tracked for >12 weeks, 10 moved >100 km in one or more weeks. Median cumulative displacement distances over 1, 10 and 30 weeks were 0 km, 260 km and 821 km, respectively. On average, over increasing time-periods, one additional roost site was visited for each additional 100 km travelled. These findings explain why culling and relocation attempts have had limited success in resolving human-bat conflicts in Australia. Flying-foxes are highly mobile between camps and regularly travel long distances. Consequently, local control actions are likely to have only temporary effects on local flying-fox populations. Developing alternative methods to manage these conflicts remains an important challenge that should be informed by a better understanding of the species’ movement patterns.

## Introduction

Many flying vertebrates can travel relatively long distances in a few days [1]–[5]. For some species, long-distance movement provides access to widely spaced and temporally variable food resources, as well as the opportunity to use different areas for roosting and for feeding. Frequent long-distance movements create particular management and conservation challenges for the species concerned, because impacts or management actions in one place may have outcomes elsewhere in the species’ range.

The fruit-bat or flying-fox family (Pteropodidae) contains many highly mobile species capable of strong flight. Flying-foxes feed at night on nectar and/or fruit, and roost during the day, often in large aggregations [6], [7]. Their patterns of movement and roost usage are, in most cases, poorly understood [6]–[8], even though a number of flying-fox species are the subject of conservation or management programs [8], [9]. Radio-tracking and limited satellite telemetry studies have revealed complex patterns of movement of flying-foxes between roosts, including long-distance movements in some species [4], [10]–[14].

The grey-headed flying-fox *Pteropus poliocephalus* is a large bat endemic to eastern Australia (average weight 782 g) [15]. Radio-tracking and satellite telemetry studies of this species have documented a range of movement patterns from long-distance nomadic movements to short ranging behaviours [2], [10]–[12]. Although predominantly feeding on native blossom and fruits, *P. poliocephalus* also raids commercial fruit crops [16], [17]. The species has been implicated as a vector of several emerging zoonotic viruses of livestock and humans (e.g., Hendra virus and Menangle virus [18]–[20]). It often roosts in urban settings, causing residents to object to its noise and smell, and become anxious about perceived risks of disease [21], [22].

For decades, various social or economic interest groups have undertaken or vigorously advocated for management actions aimed at reducing local populations of *P. poliocephalus*, either through direct culling or by inducing animals to move elsewhere, through ‘dispersal’ or ‘relocation’ of roosting colonies [16], [23]–[25]. These practices are based on the assumption that individuals frequent a particular locality or region, and can be controlled by actions undertaken at that scale. Such practices are unlikely to reduce local numbers if the species is highly mobile, unless the entire population is dramatically reduced. Such a reduction would not only require substantial effort, but would also run contrary to international and national conservation priorities [7], [26], [27].

Effective management of flying-foxes requires a good understanding of the frequency and extent of movements among roost sites. This study quantifies the patterns of movement by *P. poliocephalus* among roost sites at a range of temporal and spatial scales. Data collected from 14 satellite-tracked individuals over a period of nine months and an area of 137,600 km^2^ were used to address the following questions: (1) What patterns of movement (in terms of distances and directions) are exhibited by *P. poliocephalus* at time scales of days, weeks, and months? (2) How much do these patterns vary among individuals? (3) What is the relationship between individuals’ movement patterns and their use of different roost sites? (4) What are the implications of these findings for management?

## Methods

### Ethics Statement

Fieldwork was conducted under Griffith University Animal Ethics Committee permit AES/17/06/AEC and the Queensland Department of Environment and Resource Management Scientific Purposes permit WISP04268207.

### Study Species

*Pteropus poliocephalus* is endemic to coastal eastern Australia, with a distribution extending from central Queensland (21°S) to Melbourne, Victoria (38°S) [28]. Daytime roost sites of this species often comprise thousands of individuals and occur in rainforest, riparian, mangrove or wetland forests [10], [16], [29]. Some roosts are known to have been used for decades [16], [29]. The numbers of bats within a roost can fluctuate widely over time, associated primarily with variation in the availability of flower and fruit resources [10], [30], [31]. Large aggregations are associated with flower pulses from nectar-rich species, primarily eucalypts [10], [11], [30]. At night, *P. poliocephalus* typically feed on blossoms and fleshy fruits of trees within 20 km of their roosts, although distances between roosts and feeding sites may be as much as 50 km; the species utilises remnant forest, patches of vegetation on cleared land and urbanised areas [10], [29].

### Study Area and Capture Sites

Study animals were captured in October 2007 and June 2008 at four roost sites in south-east Queensland (SEQ): Canungra (28.0°S, 153.2°E), Stafford (27.4°S, 153.0°E), Fraser Island (25.4°S, 153.1°E) and North Stradbroke Island (Dunwich) (27.5°S, 153.4°E). Both the Canungra and Stafford roosts are occupied most months of the year and have a long history of use by *P. poliocephalus*, including use as maternity colonies, although numbers fluctuate within and between years [32, and data held by government agencies and the authors]. At the time of trapping (October 2007), both roosts had approximately 10,000 bats present. Fraser and North Stradbroke Islands are irregularly used roosts, typically occupied in the autumn and winter months of certain years in response to prolific flowering of nectar-rich trees. At the time of trapping (June 2008), both these roosts contained >50,000 *P. poliocephalus*.

### Satellite Telemetry

Fourteen adult male *P. poliocephalus* were fitted with satellite transmitters and released at the capture site: in October 2007, two bats from Canungra and two from Stafford, and in June 2008, five bats from Fraser Island and five from North Stradbroke Island. Flying-foxes were captured using mist-nets (mesh size 31 mm) suspended between 11 m tall poles, set in the early morning and late evening in the flight path of animals leaving or entering the roost site. Inhalation anaesthesia (Isoflurane, Laser Animal Health Pty Limited) was used to sedate animals for attachment of the transmitter and collar, following the protocol described by Jonsson *et al.* [33]. Males were selected to minimise the impacts on bats of carrying the transmitters: males are larger than females and are not subject to stresses of pregnancy and lactation.

Sixty-three male *P. poliocephalus* were caught and all were aged, weighed and assessed for general body condition. Only large reproductively mature males (weight 615–845 g, forearm length 155–171 mm) were fitted with solar-powered satellite transmitters (12 g PTT-100, Microwave Telemetry, Columbia, USA; see also [34]). Total package weighed approximately 22 g (<3.6% of the body mass of the smallest individual); transmitters were fitted around the neck using a soft leather collar lined with 3 mm neoprene; oriented crosswise with the solar array facing upward in roosting animals to reduce recharge time, improve power, and enhance accuracy (see Figure S1). Transmitters were programmed to a duty cycle of 10 hours on, alternating with 48 hours off, which provided sufficient off time to recharge the battery and provided regular location data including positional fixes during both day and night. The minimum time between two consecutive roost fixes was therefore 48 hours (with one recorded exception). The theoretical maximum number of fixes per 10-hour transmission cycle in the study area was 14, with accuracy and success dependent on the level of battery power. This duty cycle enables analysis of movements both between day roosts and during nocturnal feeding, although only the former are considered here. Data were obtained via the Argos Global Data Collection and Location Service System (Collecte Localisation Satellites, France). Each data record (fix) included the latitude and longitude, presumed accuracy of the location (in seven classes), UTC time and date, and battery voltage.

### Data Manipulation and Analysis

The raw transmission data from all 14 tagged flying-foxes comprised 4,356 location records, of varying presumed accuracy. Records for roosting sites were extracted using fixes that were either: (1) obtained during daylight hours (between sunrise and sunset, data from the Geoscience Australia website adjusted for geographical location); or (2) cases where bats had returned to known roosts before sunrise. From the resulting 2,616 roost records, we removed the 14% which fell within Argos accuracy classes A, B and Z, with no upper limit to their error margins, yielding 2,254 fixes for further analysis.

An average of 4.5 fixes were recorded per 10 hour ‘on’ cycle (range 1–11, SD 1.1–2.4). These were mapped in ArcView 3.3, and manually scrutinised by overlaying geographical landmarks and known roost locations [35, and data held by the authors and management agencies]. When large parts of the ‘on’ duty cycle fell during daylight hours, roost locations were visible as clusters of fixes, generally within about a 2 km radius. These were reduced to one roost fix per bat per day as follows. Fixes that were within 2 km of a known roost location (N = 48) were adjusted to match its precise location. Away from known roost sites, potential ‘unknown’ roosts (N = 29) were recorded if there were at least two daytime records less than 2 km apart, and the likely exact roost site was then sought by examination of aerial photographs, field inspection or discussions with local naturalists, wildlife carers and biologists. When such roosts could not be ground-verified, the fix with the best accuracy rating was used; three unverified roosts were clearly visited by two or more tagged bats, whereas the other 17 involved only one tagged individual. In nine of these cases the fix-clusters suggested that a single bat was using a temporary day roost away from established colonial roosts. All functionally impossible fixes were also removed by this process (163 records, i.e., 7%, including those in the ocean far from the coast or vast areas of treeless land). The resulting working data set comprised 2,091 roost records with Argos presumed accuracy other than A, B and Z (<1.5–5 km), 1,045 of which had a presumed accuracy class of 3 or 2 (<0.5 km). Each roost site was given an identifying name.

The number and duration of visits by each individual to each roost (counting revisits) were calculated from data on ‘roost visits’, defined by the dates of first and last consecutive fixes from a particular individual recorded at a particular roost site. The visit duration was the elapsed number of days at that roost (including some durations of one day only). During the 48 hour ‘off’ duty cycle, there was no transmission, which caused regular data gaps of 2–3 days, and hence the roost visit durations included these gaps. In 20 cases, there were transmission gaps of >5 days within sequences of consecutive records at the same roost; these were considered unreliable data and were removed prior to analysis. Revisits to previously used roosts were counted separately; these occurred if an individual had meanwhile been recorded at other roost sites. We documented 161 separate roost visits by the 14 flying-foxes, from which the frequency distribution of roost visit durations was obtained. All roost sites used by more than one animal were further examined to see if there was synchrony in their arrival and departure dates. We also calculated the maximum step sizes (distances between consecutive useable fixes at different roosts) across each individual’s full tracking duration, and the frequency distribution of step sizes pooled across all individuals but omitting 22 cases in which consecutive roosts were separated by transmission gaps of >5 days. To provide a time-standardised estimate of short-term inter-roost movements, we additionally calculated minimum, medium and maximum step sizes for each individual, and the step size frequency distribution across all individuals, using only those cases where consecutive different recorded roosts were two days apart (the minimum time provided by the transmitter duty cycle).

We used sampling units of ‘timed movements’ to analyse distances travelled and numbers of roost sites used during fixed time-periods of approximately 1, 5, 10, 20 and 30 weeks. To calculate movements for approximate 1-week periods, each individual’s roost records were given a repeating number sequence between 1 and 7 to flag the day of each successive week between the dates of capture and last usable position fix. Particular weeks were selected for analyses if there was a valid roost record for day 1 together with either day 6, 7 or 8 plus at least one record in between. On 8% of all occasions there was no record for days 6, 7 or 8, and the next closest record was used (day 5 or 9). There were no records between the first and last day in 5% of these cases which were removed from the analysis, providing a sample of 333 weekly records, of which 87% had at least two records in between first and last days. To calculate movements for 5 weeks, records were collapsed into as many periods of five consecutive weeks as possible using the criterion that each consecutive week had sufficient data (as defined above). Weeks without sufficient data were omitted, creating gaps between usable sequences. This process was repeated independently for time-periods of 10, 20 and 30 weeks.

Three measurements were calculated for each timed movement period: net displacement (the shortest path between an individual’s first and final roost sites in a time-period); cumulative displacement moved (the sum of all recorded distances between consecutive roost sites); and the number of different roosts used. The latter two measurements are minimum estimates due to possible data gaps during the transmitter off period or when accurate fixes were not obtained. We analysed the frequency distributions of the net and cumulative displacements, and the latter’s relationship with the number of roost sites used, across different time-periods. We calculated each individual’s maximum displacement, first as the maximum distance between any pairwise combination from all roosts visited during any 10-week period recorded for that individual, and second across each individual’s full tracking duration. The maximum step size (as defined previously) within a 10-week time span was further calculated using all 10-week time-periods available for each individual.

Information on the regional flowering patterns of tree species that are important in the diet of *P. poliocephalus* [11], [36] between October 2007 and April 2009 was obtained by maintaining regular contact with apiarists, as well as through direct observations by the authors and discussions with naturalists and ecologists.

## Results

### Long-term and Long-distance Ranging Behaviours

The 14 male *P. poliocephalus* were satellite-tracked for a range of 13–277 days (average 175 days) during which roost location data were received for 8–154 days per individual (average 78 days; Table S1). The total number of roost fixes received with an Argos presumed accuracy of <1.5 km ranged from 9–324 per individual (average 149: Table S1); half the fixes had a presumed accuracy <0.5 km (averaging four useable fixes every two days; range 0–6 fixes).

**Figure 1:**
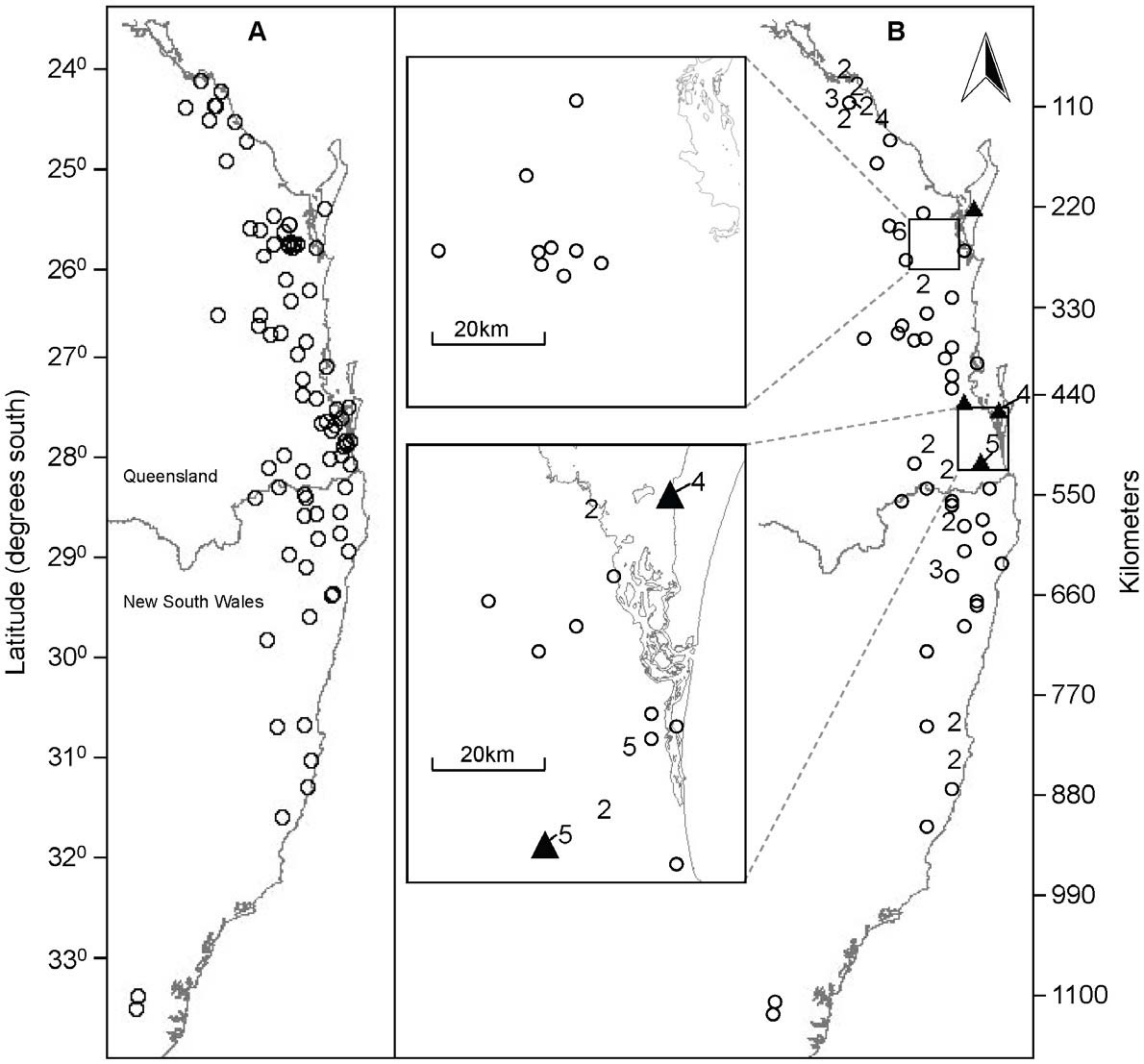
Roosts used by 14 satellite-tracked *P. poliocephalus* between October 2007 and April 2009. A. Location of the 77 roosts used during the study. B. Map of roosts showing the number of tracked individuals known to have used each roost, excluding the capture date (open circles  =  roosts used by one individual only). Triangles represent the roosts where individuals were captured.

**Figure 2:**
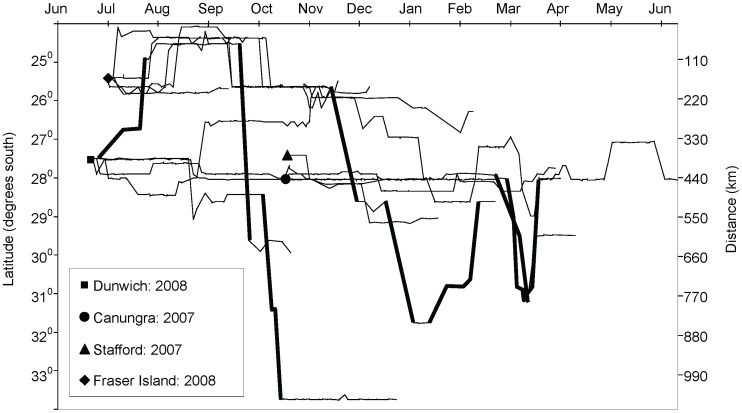
North-south movement patterns of 13 satellite-tracked *P. poliocephalus* from their points of capture. Only individuals with 12 or more weeks of data are presented. Legend shows the location and year of capture. Long-distance movements (net displacement >300 km per month) are highlighted in bold. East-west movements are not shown.

**Figure 3:**
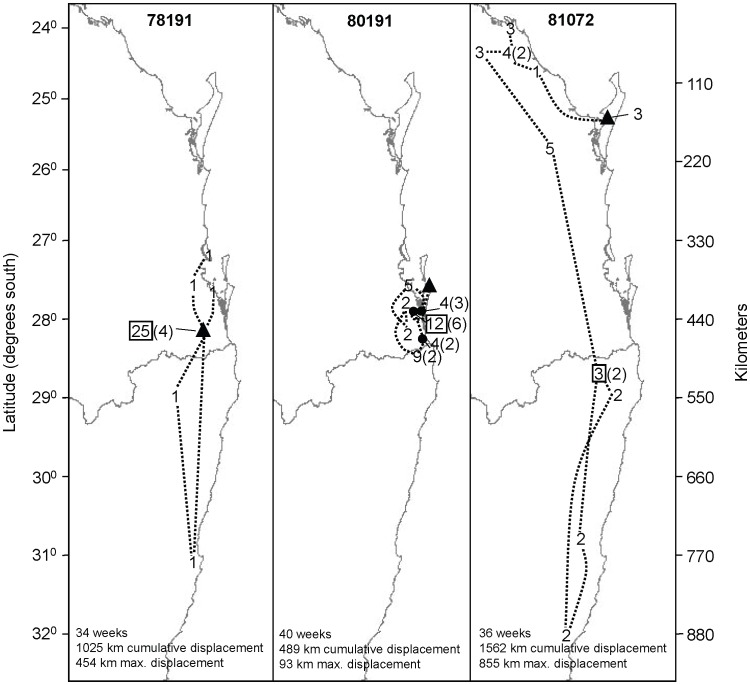
Weekly movement patterns of 3 satellite-tracked *P. poliocephalus* from their points of capture. Triangles show the points of capture. Map shows the individuals’ identification numbers (see Table S1), their roost locations at each week’s start and the numbers of weeks spent at each roost (with number of separate visits if >1 in brackets); squares represents end points. For clarity, other roosts visited within-weeks are not shown. Summaries at bottom show number of transmission weeks, cumulative displacement and maximum displacement.

The recorded roost locations of all individuals spanned a north-south distance of 1,075 km and an east-west distance of 128 km (Figure 1). Twelve of the 14 tagged individuals had moved from the point of capture within three weeks (Figure 2). Distances and directions travelled varied greatly, within and between regions (Figures 2 and 3). Five individuals travelled overall cumulative displacement distances >1,000 km (Table S1). The three individuals with the longest tracking durations (254–277 days) had maximum overall displacement distances of 93, 547, and 855 km, whereas their respective net displacements from first to last roost fix were 33, 312, and 378 km, and the corresponding cumulative displacements were 489, 1652, and 1562 km (Figure 2, Table S1). Four individuals returned to their points of capture at some time during the study (Figure 2, Table S1). Two individuals moved a step size during two days (the minimum time between consecutive day roost fixes) as large as 166 km, but if night feeding records were also considered, five individuals had 2-day step sizes of >200 km, including one of 500 km (Table S1). One animal moved 130 km within 4.8 hours (calculated using night-time fixes), which is equivalent to an average flight speed of 27 km/hr.

Individuals that achieved larger maximum displacements did so by making occasional rapid long-distance movements rather than through progressive sequences of smaller steps (Figures 2 and 3, Table S1). Overall, the flying-foxes tended to remain at the north of their range (24–28°S) during winter (June−August), and moved to higher latitudes after September (Figure 2). Five individuals undertook a total of nine long-distance movements (net displacement >300 km during one month; Figure 2), three travelled northward (from January–March and in July), and five travelled southward (from September−December and in March). Eight of the nine long-distance movements were to areas where there were reports of mass flowering of known food trees (including *Eucalyptus tereticornis, E. siderophloia, E. pilularis, Corymbia citriodora, C. intermedia, Melaleuca quinquenervia*: all Myrtaceae).

### Quantitative Patterns of Movement and Roost Site Use

A total of 77 roost sites were recorded (Figure 1). Of these, 48 (62%) had been documented prior to the present study. The remainder were mostly in remote locations to the north and west of the study area, and were used during spring. These could be previously undocumented roosts or may represent individuals or small transient groups near feeding areas. Nineteen roosts were used by two or more tagged individuals, and there were 25 occasions when two tagged individuals coincided in their roost location, although generally without synchrony of arrival or departure. Revisits to roost sites were common but variable, with one individual revisiting the same roost on eight different occasions during an elapsed time of 35 weeks. The average time between successive revisits across all individuals was 22 days (SD = 22, range 4–90, N = 61 pairs of visits).

**Figure 4:**
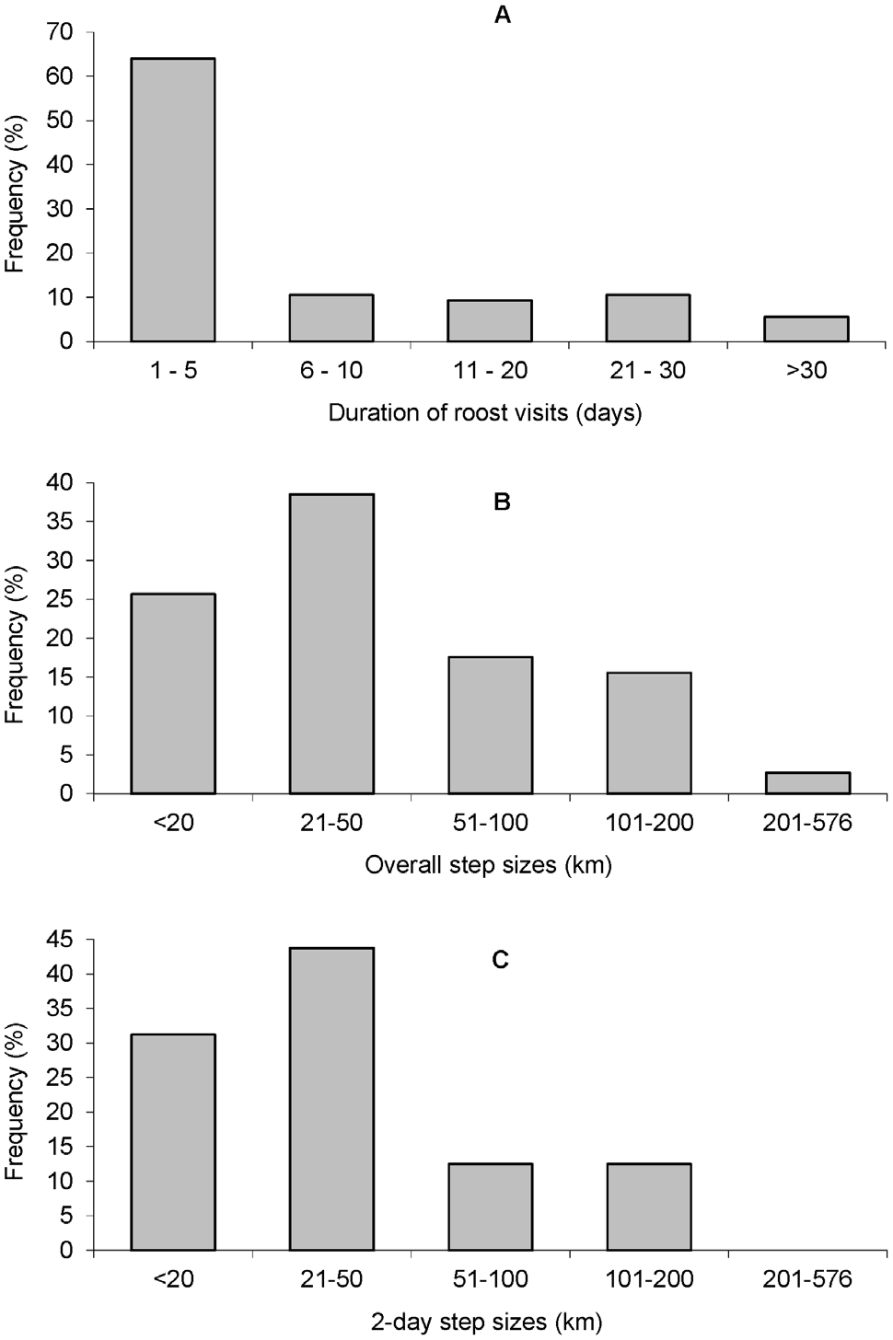
Frequency distributions of the duration of roost visits and the distances between consecutively-recorded roosts. Data from 14 satellite-tracked *P. poliocephalus*. A: Roost visit durations (the elapsed number of consecutive days at a roost, with no data gaps >5 days). Revisits were counted separately. N = 161 visits. Median roost visit duration = 2 days; mean = 9 days. B: Overall step sizes (distances between consecutive useable fixes at different roosts) across each individual’s full tracking duration, after removal of elapsed durations of >5 days; N = 148 steps. Median = 39 km; mean = 61 km. C: 2-day step sizes across standard 2-day time spans (the minimum possible under the transmitter duty cycle). N = 64 steps. Median = 32 km; mean = 45 km.

The recorded visit duration at any particular roost site was typically short, with 64% being for <5 days; however, the frequency distribution was skewed (Figure 4a). About 26% of roost visits involved >10 consecutive days (Figure 4a), but these visits accounted for 78% of the total 1,480 roost days. One flying-fox remained at its point of capture for 133 consecutive days. Extended periods at one roost were often followed by times of frequent movement among roost sites which were either in close proximity or involved progressive stops during longer north-south movements. Between consecutive recorded roosts, the flying-foxes travelled modal step sizes of 21–50 km in elapsed times of 2–5 days; the mean step size in 2–5 days was 61 km (range 3–576 km) and the mean 2-day step size was 45 km (range 3–166 km) (Figure 4b,c).

**Figure 5:**
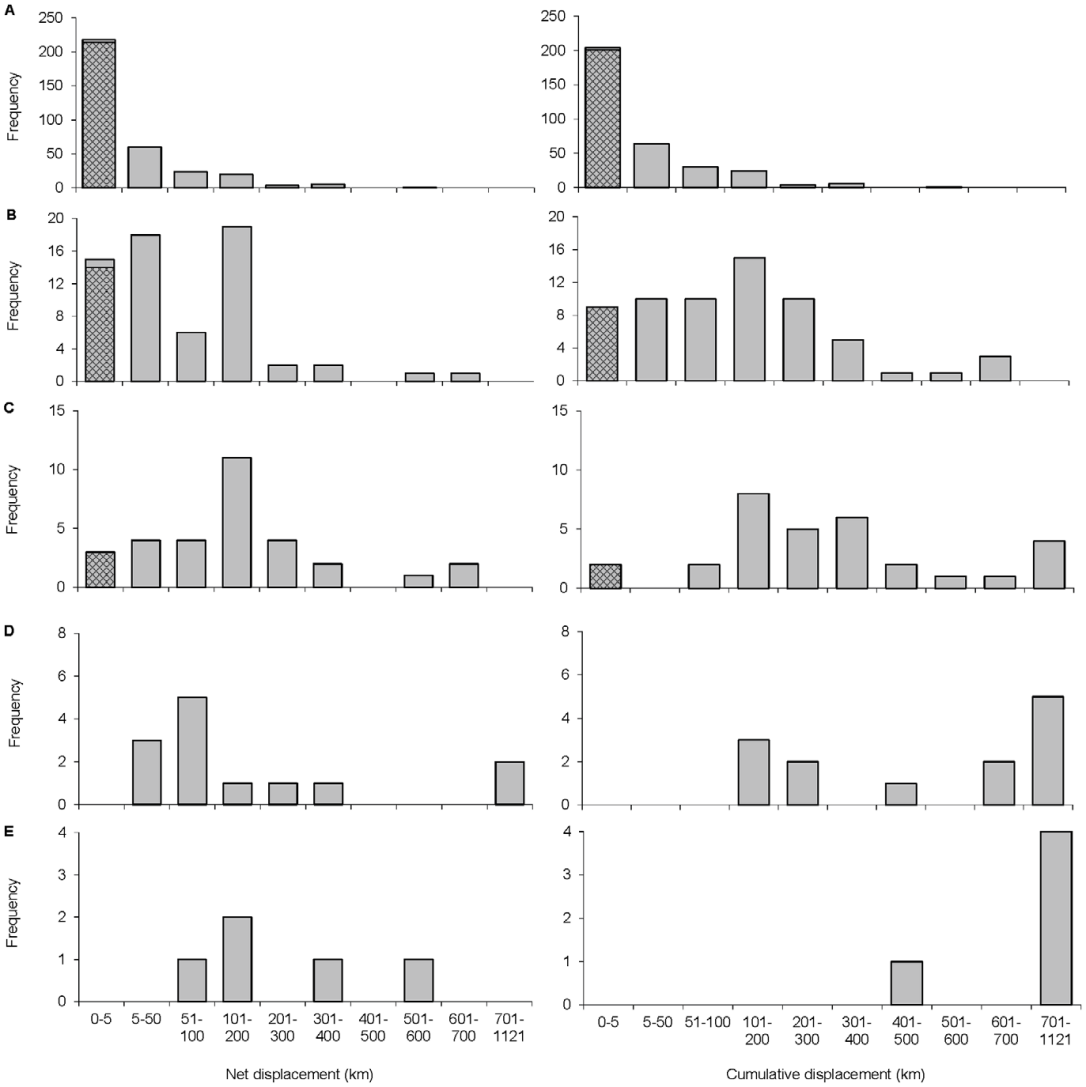
Frequency distribution of net and cumulative displacements by flying-foxes among roosts in different time-periods. A: 1 week, B: 5 weeks, C: 10 weeks, D: 20 weeks, E: 30 weeks. Net displacement is the distance separating roost sites used by an individual at the start and at the end of a time-period. Cumulative displacement is the sum of all recorded distances between consecutive roost sites for each time-period. Hatched areas show the number of zero distances in a time-period. Sample sizes as for Table 1 and Table 2.

Within one-week periods, both the net and cumulative displacements were mostly of zero km (Table 1, Table 2, Figure 5), largely due to occasional long stays by individuals at particular roosts (see above). In 6% of the 214 cases of zero net weekly movement, the cumulative displacement exceeded the net displacement indicating that these individuals had made side-trips to other roosts during the week; there may have been some additional undetected side-trips during the 48-hr transmitter off period. All individuals showed broadly similar distributions of weekly net displacement; most displacements were zero but some were >50 km and 10 of 13 flying-foxes tracked for >12 weeks had some weekly movements >100 km (Figure S2).

**Table 1:** Net displacement moved by flying-foxes among roosts in time-periods of 1–30 weeks duration.

Time-period (weeks)	N	N = 0 km	Mediankm	Meankm (SD)	Rangekm
1	333	214	0	29 (68)	0–576
5	64	14	48	93 (123)	0–626
10	31	3	147	172 (173)	0–662
20	13	0	90	208 (259)	27–776
30	5	0	158	259 (206)	94–576

Net displacement is the distance between first and last roosts during each time-period.

N: number of ‘timed movements’ in each time-period (see text).

SD: standard deviation.

**Table 2:** Cumulative displacement moved by flying-foxes among roosts in time-periods of 1–30 weeks duration.

Time-period (weeks)	N	N = 0 km	Mediankm	Meankm (SD)	Rangekm
1	333	201	0	33 (70)	0–576
5	64	9	111	161 (166)	0–690
10	31	2	260	316 (236)	0–821
20	13	0	618	584 (381)	120–1121
30	5	0	821	798 (217)	489–1059

Cumulative displacement is the sum of all sequential distances between consecutive recorded roost sites during each time-period.

N: number of ‘timed movements’ in each time-period (see text).

SD: standard deviation.

The maximum value for net displacement did not vary greatly among the five time-periods (range 576–776 km), however, the medians, means and modes of both net and cumulative displacement increased greatly from one to 10 weeks whereas from 10 to 30 weeks this rate of increase was maintained only in cumulative displacement, whilst the net displacement increased more slowly (Table 1, Table 2, Figure 5). For example, at one week, the median and mode were all zero for both net and cumulative displacement, compared with median net and cumulative displacements of 147 km and 260 km respectively at 10 weeks, increasing to 158 km and 821 km at 30 weeks (Table 1, Table 2). This pattern suggests that a maximum latitudinal seasonal displacement had generally been reached within less than 30 weeks, although there were continuing regional and local movements at a scale of tens of km within a few weeks by all flying-foxes (see also Figure 2). Among the 13 individuals with >12 weeks of data, the range of maximum displacements was 35–679 km at 10 weeks and 63–855 km over the whole study (Table S1). A lack of zero net distances in histograms for longer time-periods (Figure 5) indicates low medium-term fidelity to individual roost sites over periods of several months.

**Figure 6:**
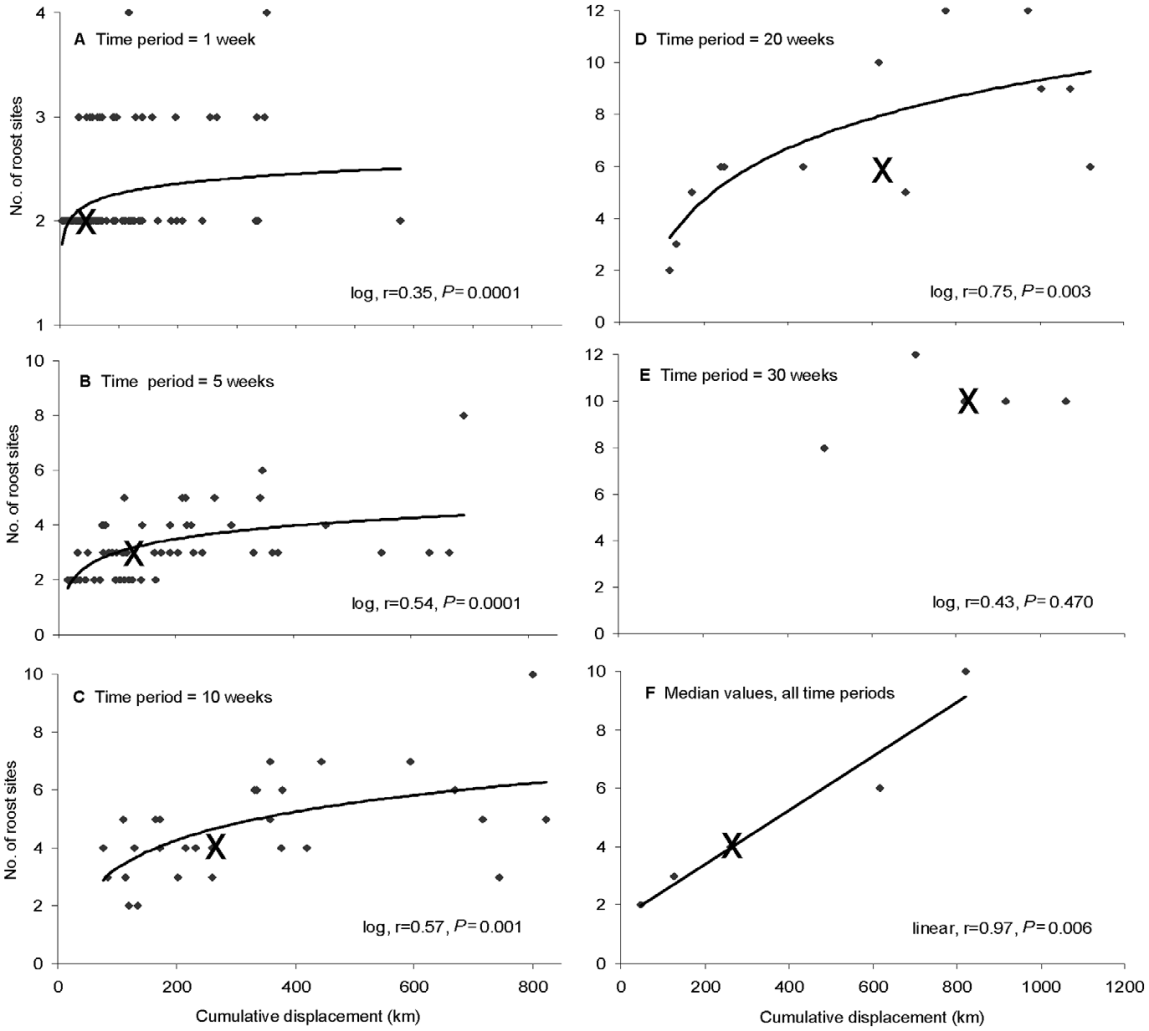
Number of roosts used by flying-foxes in relation to cumulative displacement during different time-periods. A: 1 week (N = 132), B: 5 weeks (N = 55), C: 10 weeks (N = 29), D: 20 weeks (N = 13), E: 30 weeks (N = 5). ‘X’ shows the median of each dataset. F graphs the median values of each dataset. Cases where flying-foxes did not shift roosts during a time-period were excluded. Fitted lines show significant regressions (*P*<0.05), with either log or linear selected as the best fit based on Pearson’s r values. Note that the results in A were unaffected by removal of the isolated point at displacement of 600 km.

For cases where flying-foxes shifted roosts at least once during a time-period, the number of different recorded roost sites increased logarithmically with the cumulative displacement within each time-period up to 20 weeks; for 30 weeks where N = 5 there was no significant relationship (Figure 6). The logarithmic relationships confirm that, while individuals used more roosts as they progressively travelled further, the longest travel distances were achieved by increasing the step size between roosts. Between time-periods the relationship was linear: as elapsed time increased, flying-foxes increased the number of different roosts visited in proportion to the distance travelled, adding about one additional roost for each accumulated 100 km travelled, which on average occurred every 3–4 weeks although there was very large variation (Figure 6, Table S1).

## Discussion

### Patterns of Movement and Roost Site Use by *P. poliocephalus*

This study has tracked the broad-scale movement patterns of *P. poliocephalus* at a finer temporal resolution than has been previously possible, due to improvements in lightweight satellite telemetry. Our findings agree with previous work that Australian flying-foxes are highly mobile, capable of frequent movement among roost sites in a regional area as well as long-range movements [10]–[12], [37]. The statistical quantification of the species’ movement patterns shows that (i) individuals move among roost sites far more frequently than previously reported; (ii) individuals are capable of rapid sustained long-distance flight and (iii) movements vary considerably between individuals and within individuals over time. These results are consistent with previous findings that *P. poliocephalus* exists as a single, genetically panmictic population across its range [37]–[39].

Previously suggested characterisations of movements by *P. poliocephalus* include annual north-south migration, annual return to the place of birth, and dispersal into small groups during winter [12], [16], [29], [40]. Our results support the proposal that *P. poliocephalus* exhibits a range of movement patterns, from residency to regional and long-distance nomadism, combined with facultative latitudinal migration [2], [11]. Our data revealed some seasonally-correlated long distance movements of several hundred kilometres, consistent with latitudinal migration, although only for a subset of individuals. Like their avian counterparts, Australia’s flying-foxes display movement patterns that respond to the largely irregular and ephemeral resource shifts characteristic of the continent [10], [11], [41]. Indeed, it is becoming increasingly apparent that patterns of large-scale movement in many animal species, particularly in the southern hemisphere, are more variable and complex than the regular north-south movements considered typical among long-distance migrants of the northern hemisphere [1], [5], [41].

Our observation that most long-distance movements in this study were to locations where there was intensive flowering of known food species is consistent with the findings of previous studies [10], [11], [30]. The high variability between individuals and overall complexity of movement patterns evident in our data are consistent with the view that changes in the distribution of feeding opportunities primarily drives migration in this species [10], [11], [30]. The plant species associated with long-distance movements in this study flower seasonally, however, these flowering patterns are complicated by very high inter-annual variability and spatial heterogeneity [31], [36].

Local and regional movements among roosts by *P. Poliocephalus* (at scales of tens of kilometres) would arguably be most likely to occur in situations where a diversity of forest types allows year-round access to food resources within a single region [10], [11], [42], [43]. The results of this study support this proposition. Subtropical eastern Australia at about 25°–30°S contains a high diversity of vegetation types, including high abundances of many different species of nectar-rich trees and shrubs, which flower in all months of the year [44], [45]. This is also the region where the highest population densities of *P. poliocephalus* occur [28], [31] and where the greatest concentration of roosts and local movements were observed in this study.

There was regular interchange of individual *P. poliocephalus* among roost sites within a region, with individuals often remaining less than five days at a roost. Even irregularly-used roosts remained occupied for much longer periods of time than they were used by individual animals. Short-duration stays may be a response to local shifts in food availability, or may assist individuals to obtain information about food resources, or improve social (e.g., mating) opportunities. Additionally, some roosts were clearly stopovers for animals undertaking longer movements. However, extended stays of months at a single roost were also observed. Individual flying-foxes have been reported roosting at a single site for periods up to seven months, in response to a reliable food source nearby [10], [11], [42]. In general, proximal cues for flying-fox movement and the causes of variation in their movement patterns remain poorly understood.

### Comparison with Other Flying-foxes

Detailed information on the movements of other Australian flying-fox species is limited, although there are reports of net movements of 486 km in 90 weeks for *P. scapulatus* [46] and 249 km in 49 weeks for *P. alecto* [14]. Most flying-foxes within the Asia-Pacific region have not been well studied or systematically monitored. In Asia, *P. vampyrus* has been reported moving 363 km in 4 days [13], while *Eidolon helvum* in Africa has been reported moving 370 km in one night [4]. Flying-foxes within the Asia-Pacific region are often viewed as pests of commercial fruit crops or nuisances to the tourist trade, and culled at orchards and roosts (e.g., *P. giganteus, P. hypomelanus*, *P. tonganus*) [7], [47], [48].

### Caveats to this Study

All the data obtained in this study were from adult male *P. poliocephalus*; potential differences between sexes may warrant examination. In previous studies of *P. poliocephalus*, females have displayed the same general movement patterns as males [10], [11]. However, lactating females of the ecologically similar black flying-fox (*P. alecto*) have been reported to travel greater distances between roosts and foraging sites than males [49].

Another caveat is that error estimates of location fixes in satellite-tracked flying-foxes can be considerably larger than those indicated by Argos (typically around 2.5 km for location class 0, and mainly up to 2 km for classes 1–3) [50]. We addressed this issue in three ways: first, by using a collar design which enhanced signal strength and therefore accuracy (see Methods and Figure S1); second, by using the clusters of within-day repeat fixes to identify roost locations; and third, by cross-referencing fixes with independent information on the locations of known roosts. In any case, a somewhat higher level of location error would not affect our substantive conclusions about movement patterns at the spatial scales studied here. Furthermore, our conclusion that individuals move frequently between roosts is conservative, as some roost shifts may have remained undetected during the 48-hour transmitter ‘off’ periods.

### Management Implications

*Pteropus poliocephalus* and other flying-fox species inhabiting Australia (*P. alecto*, *P. conspicillatus, P. scapulatus*) are the target of community concerns in relation to public health, amenity and impacts on agriculture. Since European settlement of Australia, people have taken or advocated various actions aimed at reducing flying-fox numbers or moving ‘problem’ roost sites [9], [16], [51]. Until 1986 in New South Wales and 1992 in Queensland, all flying-fox species were listed in government statutes as agricultural pests in need of control [52]. Public calls for culling have re-emerged in recent years following the identification of a new and highly virulent zoonotic disease, Hendra virus, which may be transmitted to horses by flying-foxes [19], [24].

However, since the 1990s, the conservation significance of flying-foxes in Australia has been increasingly recognised. *Pteropus poliocephalus* is listed as ‘Vulnerable’ by national and state governments and under the international IUCN Red List [9], [53]. Protection and listing have occurred in response to large declines in abundance resulting from the combined effects of habitat destruction, agricultural control efforts, competition with *P. alecto* and disturbance at roosts, and have been spurred by recognition of the flying-foxes’ important ecosystem functions as pollinators and seed dispersers of many tree species [26], [27].

Despite such legislative protection, the use of lethal control measures to protect fruit crops and the harassment of flying-foxes at day roosts have continued, both legally (under permit issued by state wildlife management agencies) and illegally. These attempts at relocation and local control have ignored the capacity of flying-foxes to conduct frequent long-distance movements. This study has provided new data which support previous suggestions that culling cannot be a practical tool for reducing the impacts of flying-foxes, whether these comprise crop damage or disease risk [9], [31], [54]. Culling of individuals can achieve only short-term population reduction at a local scale, since flying-foxes can easily and rapidly re-occupy culled areas, and are especially likely to do so if there is nearby flowering of nectar-rich trees. Indeed, during more than a century of culling flying-foxes in Australia and a longer-term reduction in their overall population size, the incidence of damage to fruit crops appeared unchanged [16], [17], [55]. High mobility means that the effects of local culling will be diluted across the entire population of the species. For culling to be effective, it would need to be mounted on a sufficiently large scale to reduce the species’ entire population size. Such an approach would be contrary to the conservation priorities discussed earlier.

Public demands for roost relocations have also increased over the past two decades, concurrently with rapid increases in urbanisation in the coastal subtropics [24]. The results of this study further suggest that roost relocation is unlikely to have the desired goal of permanently shifting a local flying-fox population from an area. As in the case of culling actions, even if relocation activities do induce flying-foxes to move away in the short term, empty roosts are likely to be reoccupied during subsequent months or years or, alternatively, new roost sites may be established nearby. Indeed, an analysis of 10 case studies concluded that actions to prevent roosts from being re-established typically need to be repeated many times within and between years [25]. For example, 12 years of repeated noise disturbances did not prevent flying-foxes from regularly returning in large numbers to a roost in one rural township, while there was a concurrent establishment of additional new urban roost sites in the area [25], [56].

### Conclusions

Quantitative analysis of the movement patterns of *P. poliocephalus* leads to the conclusion that most control actions are likely to have only temporary effects on local populations. These data explain why both culling and relocation attempts have had limited success in resolving conflicts between humans and flying-foxes in Australia, despite both approaches having been utilised since European settlement. Developing alternative methods to manage these conflicts in a manner informed by a better understanding of species’ movement and roost usage patterns remains an important challenge.

The data obtained in this study have improved our understanding of the frequency and complexity of movements of *P. poliocephalus*, and enabled clear conclusions to be drawn regarding the efficacy of current management practices. However, larger samples of individuals, preferably varying in age, sex and reproductive status, are required to provide a robust understanding of movement patterns. Further technological developments which enable lighter weight and lower cost satellite telemetry should facilitate research which extends the quantitative foundations provided by the present study to more comprehensive models of flying-fox movement patterns and their drivers.

## Supporting Information

Figure S1 **Orientation of solar powered satellite transmitters on flying-foxes.** A: Collar design 1, a 12 g solar powered PTT with the bottom of the unit mounted to the collar and the solar array facing down when the animal was roosting (Photo ©T. Holmes). This orientation did not allow sufficient recharging of the unit and therefore number and accuracy of fixes were poor. B: Collar design 2, a 12 g solar powered PTT attached to the collar on its side with the solar array orientated towards the sky with an angled antenna (Photo ©G. Bottroff). This orientation reduced recharge time and improved power and accuracy and was the primary design used in this study.
(PDF)

Figure S2 **Frequency distribution of the weekly net displacement of all 13 individuals that had 12 or more weeks of data.** A: 78189, B: 78190, C: 78191, D: 78186B, E: 81073, F: 81074, G: 80187, H: 80191, I: 80172, J: 80188, K: 80189, L: 80190, M: 80192.
(PDF)

Table S1 **Data summary: individuals’ characteristics, distances moved and use of roost sites over several time-periods, for 14 satellite-tracked** ***Pteropus poliocephalus*** **.** All distances are between day roost sites, except for the longest day-night step, which can be between day roost and night feeding sites.
(PDF)
