# Supplementary material for: Long-Distance and Frequent Movements of the Flying-Fox Pteropus poliocephalus: Implications for Management
Source: PLoS One. 2012 Aug 3;7(8):e42532. doi: 10.1371/journal.pone.0042532 (PMC3411823; doi:10.1371/journal.pone.0042532)

A Collar design 1, with solar array facing down when animal roosting. This orientation did not allow for sufficient recharging of unit.

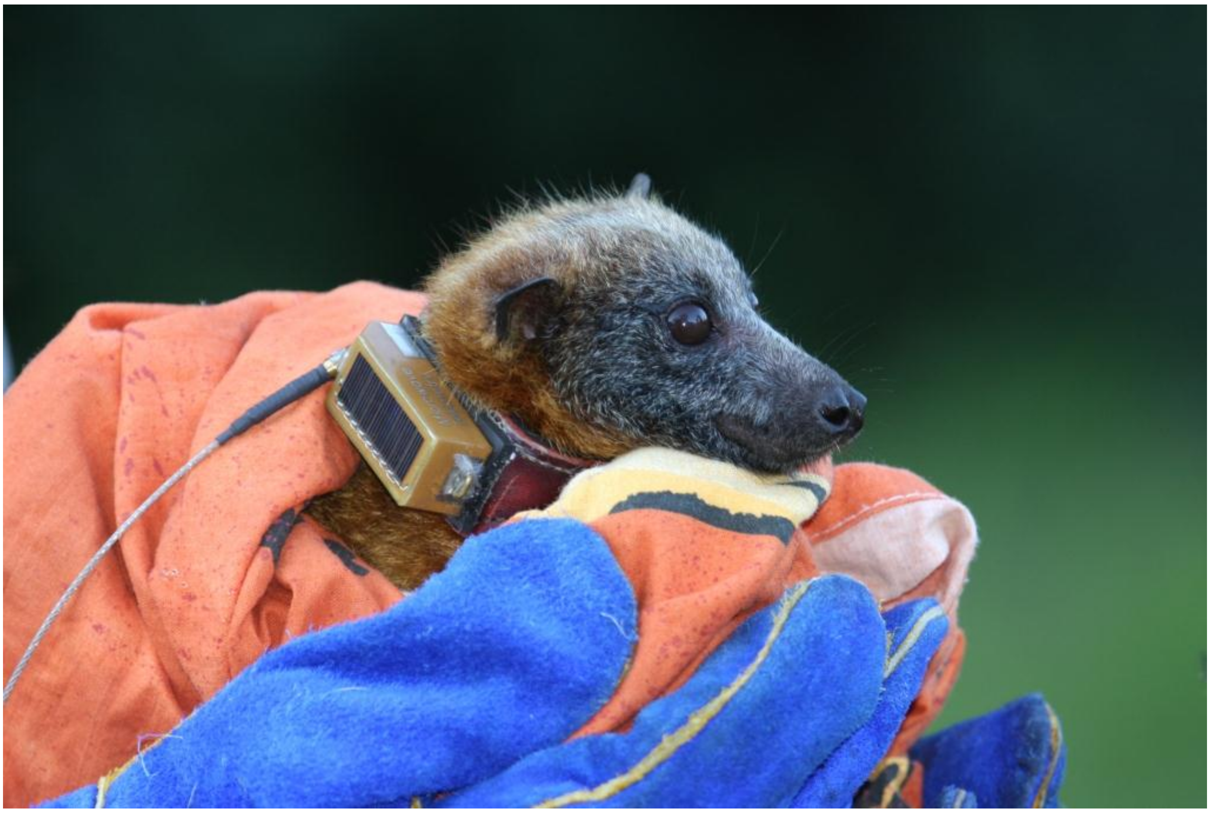

B Collar design 2, with solar array facing towards the sky when animal roosting. This orientation maintained charge and was the primary design used in this study.

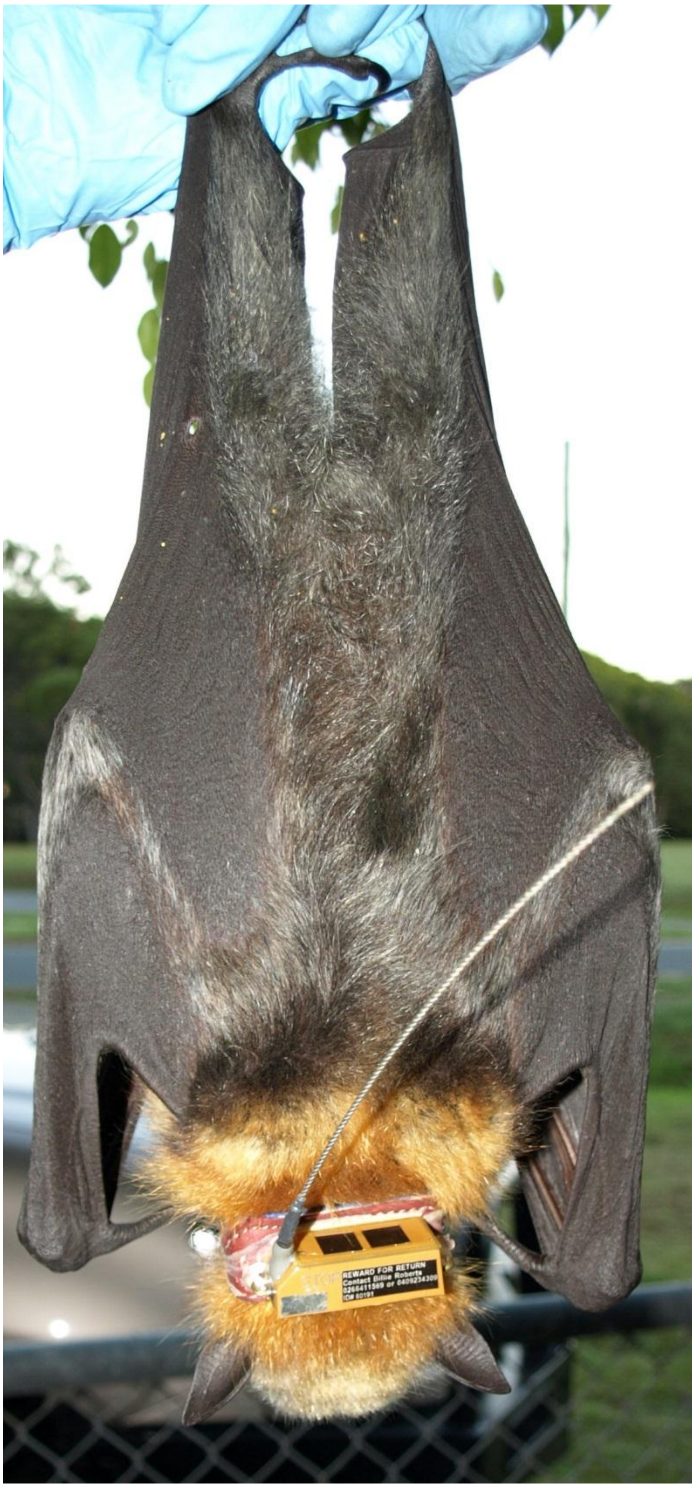

Supplement: Figure S1 — Orientation of solar powered satellite transmitters on flying-foxes. A: Collar design 1, a 12 g solar powered PTT with the bottom of the unit mounted to the collar and the solar array facing down when the animal was roosting (Photo ©T. Holmes). This orientation did not allow sufficient recharging of the unit and therefore number and accuracy of fixes were poor. B: Collar design 2, a 12 g solar powered PTT attached to the collar on its side with the solar array orientated towards the sky with an angled antenna (Photo ©G. Bottroff). This orientation reduced recharge time and improved power and accuracy and was the primary design used in this study. (PDF) [file pone.0042532.s001.pdf]
