# Supplementary figures and images for: Long-Distance and Frequent Movements of the Flying-Fox Pteropus poliocephalus: Implications for Management
Source: PLoS One. 2012 Aug 3;7(8):e42532. doi: 10.1371/journal.pone.0042532 (PMC3411823; doi:10.1371/journal.pone.0042532)

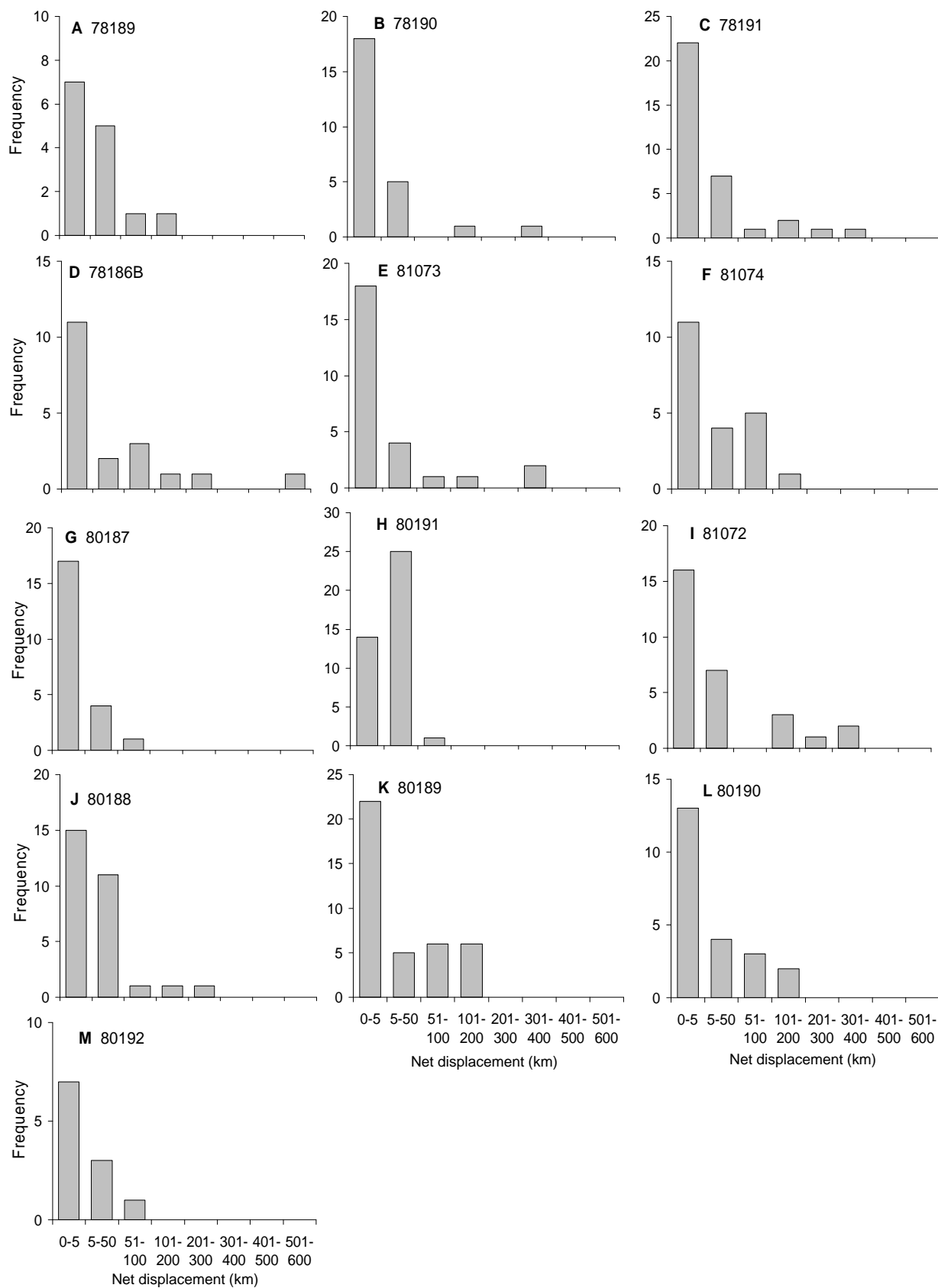

Supplement: Figure S2 — Frequency distribution of the weekly net displacement of all 13 individuals that had 12 or more weeks of data. A: 78189, B: 78190, C: 78191, D: 78186B, E: 81073, F: 81074, G: 80187, H: 80191, I: 80172, J: 80188, K: 80189, L: 80190, M: 80192. (PDF) [file pone.0042532.s002.pdf]
