# Supplementary material for: Long-Distance and Frequent Movements of the Flying-Fox Pteropus poliocephalus: Implications for Management
Source: PLoS One. 2012 Aug 3;7(8):e42532. doi: 10.1371/journal.pone.0042532 (PMC3411823; doi:10.1371/journal.pone.0042532)
Supplement: Table S1 — Data summary: individuals’ characteristics, distances moved and use of roost sites over several time-periods, for 14 satellite-tracked Pteropus poliocephalus . All distances are between day roost sites, except for the longest day-night step, which can be between day roost and night feeding sites. (PDF) [file pone.0042532.s003.pdf]

Data summary: individuals' characteristics, distances moved and use of roost sites over several time periods, for 14 satellite-tracked *Pteropus poliocephalus*. All distances are between day roost sites, except for the longest day-night step, which can be between day roost and night feeding sites.

**Flying-fox number**	**78188**	**78189**	**78190**	**78191**	**78186B**	**81072**	**81073**	**81074**	**80187**	**80188**	**80189**	**80190**	**80191**	**80192**	**Mean**	**SD**
Capture site	Stafford	Stafford	Canungra	Canungra	Dunwich	Fraser	Dunwich	Dunwich	Dunwich	Fraser	Fraser	Fraser	Dunwich	Fraser	-	-
Weight (g)	660	660	739	696	845	827	815	825	822	740	789	798	615	764	-	-
Forearm length (mm)	159	159	172	164	164	171	170	165	168	162	160	162	156	166	-	-
Date of deployment	18/10/07	17/10/07	16/10/07	16/10/07	20/6/08	30/6/08	22/6/08	22/6/08	22/6/08	30/6/08	30/6/08	1/7/08	21/6/08	30/6/08	-	-
End date	31/10/07	28/1/08	18/4/08	11/6/08	9/12/08	14/3/09	26/12/08	18/11/08	27/11/08	9/2/09	1/4/09	7/12/08	28/3/09	24/9/08	-	-
Transmission days[1]	13	101	182	235	169	254	184	146	155	219	271	156	277	84	175	75
No. of days with location data[2]	8	41	54	106	55	94	99	76	82	87	125	82	154	35	78	38
Useable roost fixes[3]	9	75	45	324	50	122	219	120	202	128	255	161	322	59	149	102
Net overall displacement km[4]	183	183	158	0	284	378	739	278	63	119	312	78	33	94	207	190
Max displacement km/days[5]	183/5	195/39	377/137	454/53	576/5	855/119	739/94	327/95	63/19	329/209	547/181	234/64	93/173	94/2		
Cumulative overall displacement km[6]	195	241	702	1025	1121	1562	1102	840	172	763	1652	980	489	194	788	491
Max. step size km in all data/days[7]	125/2	110/5	190/5	193/5	576/5	349/17	335/5	165/5	42/2	199/3	166/2	126/3	49/2	94/2		
Median step size km in 2 days/N [8]	125/1	30/1	-	51/4	-	85/2	23/3	13/10	15/3	31/7	40/9	45/9	10/14	94/1	47	36
Min. step size km in 2 days/N [9]	125/1	30/1	-	22/4	-	36/2	13/3	12*/10	15/3	18*/7	29/9	3*/9	4/14	94/1	33	38
Max. step size km in 2 days/N [10]	125/1	30/1	-	155/4	-	134/2	146/3	96/10	42/2	166/7	166/9	103/9	49/14	94/1	109	48
Longest day-night step km in 2 days[11]	125	44	230	205	500	259	336	175	42	166	166	123	49	79	179	126
N for 1-week periods	2	14	25	34	19	29	26	21	22	29	39	22	40	11		
Net displacement km/week[12]	98	21	26	32	60	53	37	31	5	24	39	27	12	17	34	23

No. roost sites used in 1 week[13]	2.0	1.0	1.3	1.4	1.0	1.4	1.4	1.8	1.4	1.4	1.5	1.8	2.0	1.4	1.5	0.3
Cumulative km in 1 week[14]	98	21	26	32	64	52	42	39	8	26	42	47	12	18	38	23
N for 5-week periods	-	2	5	6	4	6	5	4	4	6	8	4	8	2		
Net displacement km in 5 weeks[12]	-	74	51	59	259	149	150	117	16	89	105	96	21	38	94	66
No. roost sites used in 5 weeks[13]	-	2.5	2.2	2.7	2.5	2.7	3.0	4.0	2.3	2.8	3.3	3.5	2.9	3.0	2.9	0.5
Cumulative km in 5 weeks[14]	-	110	137	153	280	210	220	194	43	117	206.5	251	61	83	159	74
N for 10-week periods	-	1	2	3	2	3	2	2	2	3	4	2	4	1		
Net displacement km in 10 weeks[12]	-	195	18	114	466	301	372	127	32	169	186	147	40	72	172	136
No. roost sites used in 10 weeks[13]	-	4.0	2.5	4.3	3.5	4.0	5.0	6.5	3.0	4.7	5.0	6.0	4.3	5.0	4.4	1.1
Max displacement km in 10 weeks[15]	-	195	35	454	576	679	627	208	63	199	300	232	54	94	286	226
Max. step size km in 10 weeks[16]	-	110	35	193	576	349	335	165	42	199	166	126	46	94	187	154
Cumulative km in 10 weeks[14]	-	216	125	306	561	419	537	388	86	234	413	503	122	165	313	167
N for 20-week periods	-	-	1	1	1	1	1	1	1	1	2	1	2	-		
Net displacement km in 20 weeks[12]	-	-	27	120	333	82	742	240	64	90	165	56	41	-	178	209
No. roost sites used in 20 weeks[13]	-	-	3.0	2.0	6.0	6.0	9.0	12.0	5.0	10.0	8.5	9.0	6.0	-	7.0	3.0
Cumulative km in 20 weeks[14]	-	-	135	120	1121	436	1074	776	172	618	826	1005	245	-	593	390
N for 30-week periods	-	-	-	1	-	1	-	-	-	1	1	-	1	-		
Net displacement km in 30 weeks[12]	-	-	-	107	-	576	-	-	-	158	357.00	-	36	-	247	219
No. roost sites used in 30 weeks[13]	-	-	-	10.0	-	10.0	-	-	-	12.0	10.0	-	10.0	-	10.4	0.9
Cumulative km in 30 weeks[14]	-	-	-	919	-	821	-	-	-	703	1059	-	489	-	798	217

[1] The total number of days from the date of deployment until the unit ceased functioning or until bat activity ceased (end date).
[2] The number of days throughout the transmission period that bats were detected during daylight hours (between sunrise and sunset).
[3] The total number of fixes used for analysis; this included only securitised records with accuracy <2 km and located at a feasible roost site.
[4] The distance in km between the roost at which the individual was captured and its last recorded roost, during the lifespan of the transmitter.
[5] The furthest distance between any two recorded roosts during the lifespan of the transmitter and the no. of days between those fixes.
[6] The sum of all sequential distances between consecutive recorded roost sites throughout the study.
[7] The maximum inter-roost distance across all consecutive pairs of different recorded roosts during the lifespan of the transmitter, and the no. of days between those fixes; (total N = 167 steps across all individuals).
[8] The median inter-roost distance across all consecutive pairs of different recorded roosts that were separated by 2 days, and the no. of such steps for each individual.
[9] The minimum inter-roost distance across all consecutive pairs of different recorded roosts that were separated by 2 days, and the no. of such steps for each individual; asterisked records show movements involving temporary roosts.
[10] The maximum inter-roost distance across all consecutive pairs of recorded roosts that were separated by 2 days, and the no. of such steps for each individual.
[11] The maximum distance between consecutive useable fixes, recorded as either night feeding locations or day roost sites, which were separated by 2 days.
[12] The distance in km between the first and last roost sites during five time-periods; one week, five weeks, 10 weeks, 20 weeks and 30 weeks; for each time-period this measurement is the average across N (as shown) timed movements for each individual.
[13] The average number of different recorded roost sites during five time-periods; one week, five weeks, 10 weeks, 20 weeks and 30 weeks; for each time-period this measurement is the average across N (as shown) timed movements for each individual.
[14] The sum of all sequential distances between consecutive recorded roost sites during five time-periods; one week, five weeks, 10 weeks, 20 weeks and 30 weeks; for each time-period this measurement is the average across N (as shown) timed movements for each individual.
[15] The furthest distance between any two recorded roosts during time-periods of 10 weeks; this measurement is the maximum across N (as shown) 10-week movements for each individual.
[16] The maximum inter-roost distance across all consecutive pairs of different recorded roosts during time-periods of 10 weeks; this measurement is the maximum across N (as shown) 10-week movements for each individual.
